# Cerebrovascular and amyloid pathology in predementia stages: the relationship with neurodegeneration and cognitive decline

**DOI:** 10.1186/s13195-017-0328-9

**Published:** 2017-12-29

**Authors:** Isabelle Bos, Frans R. Verhey, Inez H.G.B. Ramakers, Heidi I. L. Jacobs, Hilkka Soininen, Yvonne Freund-Levi, Harald Hampel, Magda Tsolaki, Åsa K. Wallin, Mark A. van Buchem, Ania Oleksik, Marcel M. Verbeek, Marcel Olde Rikkert, Wiesje M. van der Flier, Philip Scheltens, Pauline Aalten, Pieter Jelle Visser, Stephanie J. B. Vos

**Affiliations:** 1 Department of Psychiatry and Neuropsychology, School of Mental Health and Neuroscience, Alzheimer Center Limburg, Maastricht University, Maastricht, The Netherlands; 2 Institute of Clinical Medicine, Neurology, University of Eastern Finland, Kuopio, Finland; 3 Neurocenter & Department of Neurology, Kuopio University Hospital, Kuopio, Finland; 4 Department of Neurobiology, Caring Sciences and Society (NVS), Karolinska University Hospital Huddinge, Stockholm, Sweden; 5 AXA Research Fund and UPMC Chair, Sorbonne Universités, Université Pierre et Marie Curie (UPMC), Paris, France; 6 Institut du cerveau et de la moelle (ICM), Hôpital Pitié-Salpêtrière, Paris, France; 7 Aristotle University of Thessaloniki, Memory and Dementia Center, 3rd Department of Neurology, “G Papanicolau” General Hospital, Thessaloniki, Greece; 8 Department of Clinical Sciences Malmö, Clinical Memory Research Unit, Lund University, Lund, Sweden; 9 Department of Radiology, Leiden University Medical Center, Leiden, The Netherlands; 10 Department of Gerontology and Geriatrics, Leiden University Medical Center, Leiden, The Netherlands; 11 Departments of Neurology and Laboratory Medicine, Donders Institute for Brain, Cognition and Behaviour, Radboud Alzheimer Center, Radboud University Medical Center, Nijmegen, The Netherlands; 12 Radboudumc Alzheimer Centre, Department of Geriatric Medicine, Radboud University Medical Center, Nijmegen, The Netherlands; 13 Department of Neurology, Alzheimer Centre, Neuroscience Campus Amsterdam, VU University Medical Center, Amsterdam, Netherlands

**Keywords:** Amyloid, Cerebrovascular disease, Alzheimer’s disease, Cognition, Neurodegeneration, Medial temporal lobe atrophy, Tau, Cerebrospinal fluid, MRI

## Abstract

**Background:**

Cerebrovascular disease (CVD) and amyloid-β (Aβ) often coexist, but their influence on neurodegeneration and cognition in predementia stages remains unclear. We investigated the association between CVD and Aβ on neurodegenerative markers and cognition in patients without dementia.

**Methods:**

We included 271 memory clinic patients with subjective or objective cognitive deficits but without dementia from the BioBank Alzheimer Center Limburg cohort (*n* = 99) and the LeARN (*n* = 50) and DESCRIPA (*n* = 122) multicenter studies. CSF Aβ_1–42_ and white matter hyperintensities (WMH) on magnetic resonance imaging (MRI) scans were used as measures of Aβ and CVD, respectively. Individuals were classified into four groups based on the presence (+) or absence (−) of Aβ and WMH. We investigated differences in phosphorylated tau, total tau (t-tau), and medial temporal lobe atrophy (MTA) between groups using general linear models. We examined cognitive decline and progression to dementia using linear mixed models and Cox proportional hazards models. All analyses were adjusted for study and demographics.

**Results:**

MTA and t-tau were elevated in the Aβ − WMH+, Aβ + WMH−, and Aβ + WMH+ groups. MTA was most severe in the Aβ + WMH+ group compared with the groups with a single pathology. Both WMH and Aβ were associated with cognitive decline, but having both pathologies simultaneously was not associated with faster decline.

**Conclusions:**

In the present study, we found an additive association of Aβ and CVD pathology with baseline MTA but not with cognitive decline. Because our findings may have implications for diagnosis and prognosis of memory clinic patients and for future scientific research, they should be validated in a larger sample with longer follow-up.

**Electronic supplementary material:**

The online version of this article (doi:10.1186/s13195-017-0328-9) contains supplementary material, which is available to authorized users.

## Background

Cerebrovascular disease (CVD) often coexists with Alzheimer’s disease (AD), and both conditions add to cognitive decline [1, 2]. The influence of coexisting CVD and AD pathology on neurodegeneration and cognitive decline in predementia stages of AD, however, remains uncertain. Understanding the role CVD pathology in early AD is key to understanding and preventing cognitive decline in AD.

In subjects with dementia, coexistent AD and CVD pathology at autopsy is associated with more rapid cognitive decline and often a more severe form of dementia than isolated AD pathology [3, 4]. A combination of AD and CVD has also been associated with a lower burden of amyloid-β (Aβ) pathology than in isolated AD [5, 6], suggesting that less AD pathology is needed for cognitive impairment in individuals who also have CVD [7, 8]. In cognitively normal subjects, it was shown that Aβ and CVD pathology are independent contributors to cognitive decline and that both increase the risk of dementia [7, 9]. Studies on the contribution of Aβ and CVD pathology on cognitive decline in individuals with subjective cognitive decline (SCD) and mild cognitive impairment (MCI) have shown conflicting results [10, 11]. Also, how each of these pathologies relates to different markers of neurodegeneration is less well understood, because previous studies point in different directions or were focused on only a single marker instead of investigating multiple neurodegenerative markers using various modalities (e.g., magnetic resonance imaging (MRI) and cerebrospinal fluid CSF)) [12–15]. Clarity regarding the relationship between coexisting AD and CVD pathology, neurodegenerative markers, and cognition will improve diagnostic and prognostic accuracy of early AD.

The aim of this study was to investigate whether in patients with SCD and MCI there is an additive effect of CVD and Aβ on neurodegeneration measured by total tau (t-tau) and phosphorylated tau (p-tau) in CSF and medial temporal lobe atrophy (MTA) visualized by MRI, as well as on cognitive decline during follow-up.

## Methods

### Subjects

Two hundred seventy-one subjects were selected from memory clinics of the single-center BioBank Alzheimer Center Limburg (BBACL; *n* = 99) cohort and the LeARN (*n* = 50) [16] and DESCRIPA (Development of screening guidelines and criteria for predementia Alzheimer’s disease; *n* = 122) [17] multicenter studies. Inclusion criteria were (1) no diagnosis of dementia at baseline and (2) baseline data available for MRI and CSF measures. When subjects participated in more than one study, we included the data from the study with the longest follow-up. The medical ethics committee at each site approved the study. All subjects provided informed consent.

### Clinical assessment

Clinical assessment included neuropsychological assessment and an assessment of medical history. Information on medical history (e.g., hypertension, diabetes, obesity) was provided by patients and/or their caregivers, or it was extracted from medical files. Neuropsychological assessment was performed according to local routine protocol of each site, including the Mini Mental State Examination (MMSE) and at least one test in the cognitive domains of memory and executive functioning. The delayed recall of a word list test was used to examine memory. For the BBACL and LeARN studies, the Rey Auditory Verbal Learning Test (RAVLT) was used [18]. For the DESCRIPA cohort, the RAVLT and Consortium to Establish a Registry for Alzheimer’s Disease word list were the primary memory tests used. DESCRIPA tests per center are described elsewhere [17]. The Trail Making Test part B (TMT-B) [19] was used to examine executive functioning. Raw scores on each test were converted to z-scores using local normative data. Z-scores below −5 (*n* = 7) were rounded to −5 to avoid bias through outliers in the data.

Diagnosis of MCI at baseline was made according to the criteria of Petersen [20]. Subjects with a z-score below −1.5 on the immediate recall or delayed recall of a word list test were classified as having amnestic MCI. Subjects with a z-score below −1.5 on any of the nonmemory tests were classified as nonamnestic MCI. Diagnosis of AD-type dementia at follow-up was made according to the criteria of the Diagnostic and Statistical Manual of Mental Disorders, Fourth Edition [21], and the National Institute of Neurological and Communicative Disorders and Stroke-Alzheimer’s Disease and Related Disorders Association [22]. Etiological diagnoses of other types of dementia were made according to standardized clinical criteria for vascular dementia [23], frontotemporal dementia (FTD) [24], and dementia with Lewy bodies [25].

### CSF analyses

CSF was collected by lumbar puncture and thereafter centrifuged and stored at −80 °C in polypropylene tubes. CSF Aβ_1–42_, t-tau, and p-tau were analyzed using the Innotest sandwich enzyme-linked immunosorbent assay (Innogenetics, Ghent, Belgium) in Gothenburg for the DESCRIPA cohort [17], in Amsterdam for the LeARN project [26], and in Nijmegen for the BBACL study [27]. To define abnormality of the CSF measures, the following predefined cutoffs were used: Aβ_1–42_ ≤ 550 pg/ml, t-tau > 375 pg/ml, and p-tau_181_ > 52 pg/ml [28].

### Genetic analyses

The apolipoprotein E (*APOE*) genotype was determined in a subgroup of the sample (*n* = 165). Assessments were performed according to routine protocol at each site, as described elsewhere [17, 29].

### MRI analyses

All subjects were scanned according to the routine MRI protocol at each site (Additional file 1). Scanning was performed at 1.0 (*n* = 14), 1.5 (*n* = 108), or 3.0 (*n* = 149) Tesla, and all scans included a three-dimensional T1-weighted gradient echo sequence and a fast fluid-attenuated inversion recovery sequence. To determine MTA, the Scheltens MTA visual rating scale [30] was used. The score on the MTA scale ranges from 0 to 4 for each hemisphere. The summed score of both hemispheres was used, where an abnormal MTA was defined using a cutoff ≥ 2 [31]. White matter hyperintensities (WMH) were measured with the visually rated age-related white matter changes (ARWMC) scale (range 0–3) [32] for the DESCRIPA cohort and with the visually rated Fazekas scale (range 0–3) [33] for the BBACL and LeARN cohorts. For the ARWMC scale, a cutoff score ≥ 2 in at least one of the measured brain areas was used to define WMH status [34, 35]. For the Fazekas scale, a cutoff score ≥ 2 was also used to define WMH status [36].

### Subject classification

To classify individuals into subgroups, we used Aβ as a measure of AD and WMH as a measure of CVD. Subjects were classified as Aβ + when CSF Aβ_1–42_ levels were abnormal. Subjects were classified as WMH+ when the WMH score was high. We created four groups based on combinations of Aβ status and WMH status: Aβ − WMH−, Aβ − WMH+, Aβ + WMH−, and Aβ + WMH+.

### Statistical analyses

We analyzed differences in clinical baseline and follow-up characteristics and neurodegeneration markers between groups using analysis of variance for continuous variables and chi-square tests for categorical variables. Prior to the continuous comparisons of biomarker values between groups (Tables 1 and 2), Aβ_1–42_, p-tau, and t-tau values were log-transformed to approximate a normalized distribution required for statistical comparisons. The raw biomarker values are shown in the tables. Comparisons between Aβ/WMH groups regarding neurodegenerative markers (Table 2) were all corrected for demographics, study, and baseline diagnosis.

**Table 1:** Comparisons of baseline and follow-up characteristics by amyloid-β and white matter hyperintensities status

	Aβ − WMH− (*n* = 140)	Aβ − WMH+ (*n* = 39)	Aβ + WMH− (*n* = 63)	Aβ + WMH+ (*n* = 29)
Baseline characteristics				
Age, years	61.7 (8.3)^a,b,c,d^	71.3 (7.7)^b,d^	66.7 (7.8)^a,c,d^	74.1 (5.0)^b,d^
Female sex, *n*	94 (67)^b^	23 (59)	32 (51)^d^	16 (55)
Education, years	10.9 (3.1)	11.9 (3.3)	11.1 (3.1)	10.3 (2.9)
Hypertension, *n* ^e^	43 (34)	9 (25)	15 (25)	9 (32)
Obesity, *n* ^e^	15 (14)	3 (11)	4 (8)	4 (21)
Diabetes, *n* ^e^	16 (21)	3 (15)	3 (7)	5 (28)
APOE ε4 allele carrier, *n* ^e^	33 (51)^a^	5 (24)^b,c^	29 (62)^a^	10 (56)^a^
Diagnosis of MCI, *n*	70 (50)^c^	21 (54)^c^	40 (64)	22 (76)^a,d^
Amnestic MCI, % within MCI group	40 (57)	15 (71)	27 (68)	17 (77)
Nonamnestic MCI, % within MCI group	30 (43)	6 (29)	13 (33)	5 (23)
CSF Aβ_1–42_, pg/ml	973.6 (312.0)^b,c^	885.0 (242.0)^b,c^	404.3 (102.6)^a,d^	419.3 (97.2)^a,d^
White matter hyperintensities^f^	0.7 (0.5)^a,c^	2.3 (0.4)^b,d^	0.8 (0.4)^a,c^	2.4 (0.5)^b,d^
Follow-up characteristics				
Follow-up duration, years	2.1 (1.5)	2.2 (1.3)	2.1 (1.2)	2.4 (1.2)
Time to progression to dementia, years	1.3 (0.5)^a^	2.0 (0.7)^d^	1.7 (0.7)	2.1 (1.2)
Progression to dementia, *n*	8 (6)^a,b,c^	9 (23)^d^	18 (29)^d^	11 (38)^d^
AD-type dementia, *n*	2 (1)^a,b,c^	7 (18)^d^	18 (29)^d^	10 (35)^d^
Vascular dementia, *n*	0 (0)	2 (5)	0 (0)	1 (3)
Frontotemporal dementia, *n*	4 (3)	0 (0)	0 (0)	0 (0)
Dementia with Lewy bodies, *n*	1 (1)	0 (0)	0 (0)	0 (0)
Dementia with unknown etiology, *n*	1 (1)	0 (0)	0 (0)	0 (0)

*Abbreviations*: *A*β Amyloid-β, *AD* Alzheimer’s disease, *APOE* Apolipoprotein E, *CSF* Cerebrospinal fluid, *MCI* Mild cognitive impairment, *WMH* White matter hyperintensities

Results are mean (SD) for continuous variables or number (%)

^a^ *p* < 0.05 compared to Aβ - WMH-

^b^ *p* < 0.05 compared to Aβ - WMH+

^c^ *p* < 0.05 compared to Aβ + WMH-

^d^ *p* < 0.05 compared to Aβ + WMH+

^e^Hypertension, obesity, diabetes, and APOE ε4 genotype were available only in a subgroup of the sample

^f^WMH measured by the Fazekas scale, range 0-3

**Table 2:** Values of neurodegenerative markers by amyloid-β/white matter hyperintensities groups

Neurodegeneration markers	Aβ − WMH− (*n* = 140)	Aβ − WMH+ (*n* = 39)	Aβ + WMH− (*n* = 63)	Aβ + WMH+ (*n* = 29)
MTA score	1.2 (1.2)^a,b,c^	2.6 (1.6)^c,d^	2.1 (1.6)^c,d^	3.4 (1.8)^a,b,d^
MTA abnormal, *n*	62 (45)^a,b,c^	32 (82)^d^	41 (67)^c,d^	26 (93)^b,d^
p-tau, pg/ml	54.5 (27.7)^b^	63.2 (29.3)	77.0 (56.3)^d^	65.2 (38.2)
p-tau abnormal, *n*	53 (38)^b^	22 (58)	45 (71)^d^	15 (52)
t-tau, pg/ml	314.7 (202.0)^a,b,c^	438.4 (248.0)^d^	499.3 (413.8)^d^	426.2 (275.2)^d^
t-tau abnormal, *n*	36 (26)^a,b,c^	20 (53)^d^	36 (57)^d^	14 (48)^d^

*Abbreviations*: *A*β Amyloid-β, *MTA* Medial temporal lobe atrophy, *p-tau* Phosphorylated tau, *T-tau* Total tau, *WMH* White matter hyperintensities

Results are mean (SD) and number (%). All analyses were adjusted for study, baseline diagnosis, and demographics

^a^ *p* < 0.05 compared to Aβ - WMH-

^b^ *p* < 0.05 compared to Aβ - WMH+

^c^ *p* < 0.05 compared to Aβ + WMH-

^d^ *p* < 0.05 compared to Aβ + WMH+

The associations between Aβ/WMH groups and changes in MMSE scores, memory, and executive functioning were assessed by slope analyses with linear mixed models. The analyses included the baseline scores and all available follow-up scores (up to 4 years). All slope analyses were adjusted for study. When the interaction between Aβ/WMH group, baseline diagnosis, and time was significant, we added baseline diagnosis as a covariate in the model. The models adjusted for baseline diagnoses are reported in the tables and figures, and the results stratified by diagnoses are reported in the text. For the MMSE, we also adjusted for age, sex, and years of education because these scores are not standardized. We also tested the influence of APOE genotype in a subgroup of the sample for whom this was available. Models were fitted with random study-specific intercept and subject-specific slopes and a first-order autoregressive correlation structure. We chose this model because it provided the best −2 log-likelihood ratio and the lowest number of parameters. Cox proportional hazards models were used to investigate the risk of progression to dementia for each group after adjusting for demographics, study, and baseline diagnosis. Statistical analyses were conducted with IBM SPSS Statistics version 24.0 software (IBM, Armonk, NY, USA) with the significance level set at *p* < 0.05. Owing to the exploratory nature of the study, we did not control for multiple comparisons. Post hoc power calculations were conducted using IBM SPSS Statistics software and the ‘simr’ package of R statistical software (version 3.3.3; R Foundation for Statistical Computing, Vienna, Austria).

## Results

### Cohort characteristics

We included 271 individuals with a mean age of 65.6 (SD 9.0) years. One hundred sixty-five (61%) were female, and 153 (57%) had a diagnosis of MCI at baseline, of whom 99 (65%) were classified as having amnestic MCI. Follow-up data were available for 233 individuals (86%). The availability of follow-up data was not different among the Aβ/WMH groups (*p* = 0.396) or studies (*p* = 0.730). After a mean follow-up of 2.5 (SD 1.2) years, 46 (17%) subjects had progressed to dementia. The majority (80%) of the individuals who progressed to dementia had a clinical diagnosis of AD-type dementia.

Table 1 shows baseline and follow-up characteristics of the four Aβ/WMH groups. The group without pathology was younger (*p* < 0.001) and progressed less frequently to dementia than the other three groups (*p* < 0.001). We found no difference in the prevalence of several vascular risk factors between the four groups (hypertension, *p* = 0.563; obesity, *p* = 0.486; diabetes, *p* = 0.106). We found no difference in Aβ load between the group with only Aβ and the group with both Aβ and WMH pathologies (*p* = 0.502). Likewise, the proportion of WMH was not different between the two WMH+ groups (Aβ − WMH+ and Aβ + WMH+; *p* = 0.175).

### Neurodegeneration markers

Table 2 shows the values and frequency of abnormal neurodegenerative markers for the Aβ/WMH groups. We found that, compared with the group without pathology, MTA was more severe in the groups with only Aβ (*p* < 0.001) and with only WMH (*p* < 0.001), as well as in the mixed pathology group (*p* < 0.001). The Aβ + WMH+ group had higher MTA scores than the group with only WMH (*p* = 0.025) and the group with only Aβ (*p* = 0.002). t-tau was increased in all three groups with a form of pathology compared with the group without pathology (Aβ − WMH+, *p* < 0.001; Aβ + WMH−, *p* < 0.001; Aβ + WMH+, *p* = 0.047), but this effect was influenced by baseline diagnosis (Aβ/WMH group × baseline diagnosis, *F* = 3.20, *p* = 0.024). When stratified by diagnosis, the effect was found only in subjects with MCI. There was no difference in t-tau levels between the Aβ − WMH+, Aβ + WMH−, and Aβ + WMH+ groups, regardless of baseline diagnosis. p-tau was increased only in the group with only Aβ compared with the Aβ − WMH− group (*p* < 0.001), regardless of baseline diagnosis. The association between p-tau and Aβ/WMH group was influenced by APOE genotype because we found that the elevated p-tau levels in the Aβ + WMH− group were limited to APOE ε4 allele carriers (Aβ/WMH group × APOE status, *F* = 3.72, *p* = 0.013).

### Baseline cognitive performance and cognitive decline

**Fig. 1:**
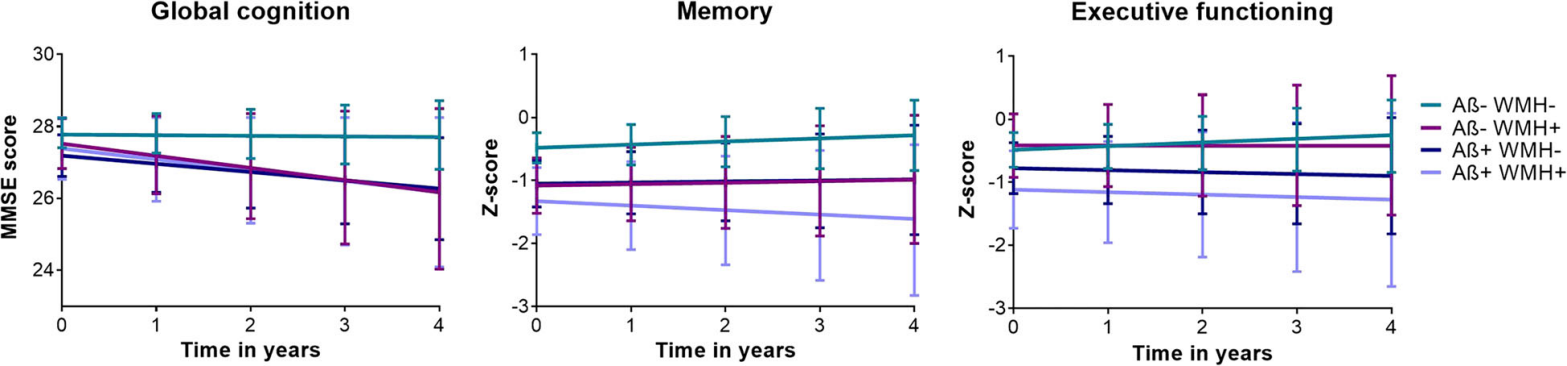
Cognitive decline by amyloid-β/white matter hyperintensities (Aβ/WMH) group for global cognition, memory, and executive functioning. The graphs show mean scores and 95% CIs of cognitive decline over time for four different groups based on Aβ/WMH status. The *left graph* shows cognitive decline for global cognition (Mini Mental State Examination [MMSE]) after adjusting for demographics, study, and baseline diagnosis. The *middle graph* shows cognitive decline for memory (delayed recall of Rey Auditory Verbal Learning Test) after adjusting for study. The *right graph* shows cognitive decline for executive functioning (Trail Making Test part B) after adjusting for study

In the total sample, MMSE scores did not differ between the Aβ/WMH groups at baseline (Table 3, Fig. 1). Baseline MMSE scores of the individuals with MCI were lower than those of individuals with SCD, regardless of pathology (*p* < 0.001). In subjects with MCI, there was a difference in baseline MMSE score only between the Aβ − WMH− and the Aβ + WMH− groups (*p* = 0.020). In subjects with SCD, there was no difference in baseline MMSE scores. In the total sample, the groups with one or both pathologies declined in MMSE score over time (Aβ − WMH+, *p* = 0.014; Aβ + WMH−, *p* = 0.035; Aβ + WMH+, *p* = 0.045), whereas scores remained stable in the Aβ − WMH− group (*p* = 0.793). The rate of decline was higher in the Aβ − WMH+ group than in the Aβ − WMH− group (*p* = 0.035). There were no differences in the rate of decline between the three groups with pathology. In subjects with SCD, the Aβ/WMH groups showed no decline over time. In subjects with MCI, the results were similar to those found in the total sample. Baseline delayed recall memory scores were lower in the three groups with pathology than in the group without pathology (Aβ − WMH+, *p* = 0.004; Aβ + WMH−, *p* < 0.001; Aβ + WMH+, *p* = 0.009), which was not influenced by baseline diagnosis. None of the groups showed significant decline over time. TMT-B scores did not differ at baseline between the groups and did not change during follow-up (Table 3, Fig. 1). APOE genotype did not influence any of the baseline or longitudinal associations.

**Table 3:** Cognitive performance and decline, by amyloid-β/white matter hyperintensities groups

		Aβ − WMH−	Aβ − WMH+	Aβ + WMH−	Aβ + WMH+
MMSE	No. of subjects	140	39	62	27
	Baseline	27.79 (27.39, 28.19)	27.52 (26.83, 28.21)	27.20 (26.62, 27.78)	27.40 (26.54, 28.25)
	Slope	−0.01 (−0.15, 0.12)	**−0.29 (−0.55, −0.02)**	**−0.22 (−0.44, −0.01)**	**−0.31 (−0.62, 0.00)**
Memory delayed recall z-score	No. of subjects	133	37	58	27
	Baseline	−0.48 (−0.72, −0.24)^b,c,d^	−1.04 (−1.48, −0.61)^e^	−1.04 (−1.41, −0.68)^A^	−1.33 (−1.86, −0.80)^e^
	Slope	0.05 (−0.03, 0.13)	0.02 (−0.12, 0.17)	0.02 (−0.11, 0.14)	−0.07 (−0.24, 0.09)
Executive functioning z-score	No. of subjects	130	37	60	24
	Baseline	−0.48 (−0.76, −0.21)	−0.41 (−0.92, 0.09)	−0.78 (−1.18, −0.37)	−1.12 (−1.73, −0.50)
	Slope	0.06 (−0.02, 0.13)	−0.00 (−0.15, 0.15)	−0.03 (−0.16, 0.10)	−0.04 (−0.23, 0.15)

*Abbreviations*: *A*β Amyloid-β, *MMSE* Mini Mental State Examination, *WMH* White matter hyperintensities

Results are mean (95% CI). Bold slope estimates = *p* < 0.05. All analyses were adjusted for study. The analyses of MMSE scores were also corrected for demographics and baseline diagnosis

^a^ *p* < 0.05 compared to Aβ - WMH-

^b^ *p* < 0.05 compared to Aβ - WMH+

^c^ *p* < 0.05 compared to Aβ + WMH-

^d^ *p* < 0.05 compared to Aβ + WMH+

### Progression to dementia

**Fig. 2:**
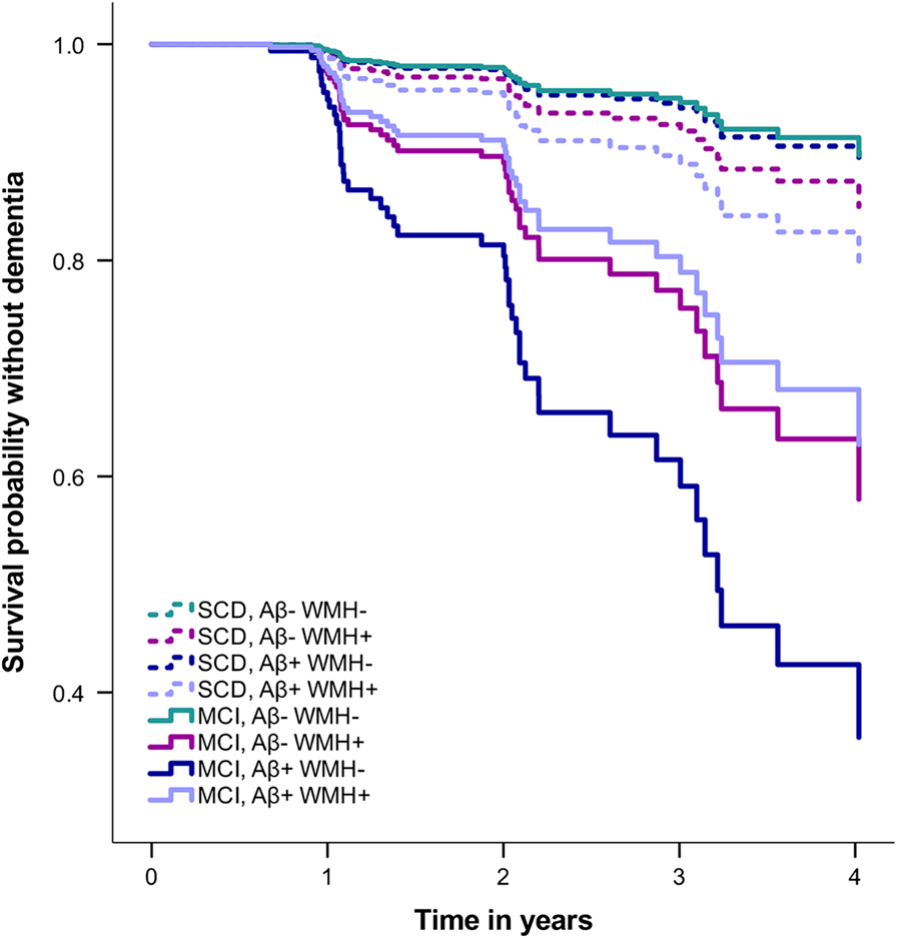
Risk of progression to dementia over time for amyloid-β/white matter hyperintensities (Aβ/WMH) groups, by baseline diagnosis. The graph shows the probability of surviving without dementia during a 4-year follow-up period for the four Aβ/WMH groups after adjusting for demographics and study, stratified by baseline diagnosis. *MCI* Mild cognitive impairment, *SCD* Subjective cognitive decline

Table 4 and Fig. 2 show the risk of progression to dementia for the Aβ/WMH groups. Compared with the group without pathology, the groups with a form of pathology have an increased risk of progressing to dementia (Aβ- WMH+ HR: 3.25, *p* = 0.021, Aβ + WMH- HR: 4.89, *p* < 0.001, Aβ + WMH+ HR:3.00, *p* = 0.036), but this was influenced by baseline diagnosis (Aβ/WMH group*baseline diagnosis: HR = 2.89; *p* = 0.007) as the effect was mainly attributable to MCI subjects (Fig. 2). There was no difference in progression rates between the groups with isolated or coexisting Aβ/WMH pathology, when analyzing the total sample or only MCI subjects. Results were similar when using progression to AD-type dementia as outcome.

**Table 4:** Risk of progression to dementia for amyloid-β/white matter hyperintensities groups

Groups	HR	95% CI	*p* Value comparisons	
Aβ − WMH−	Reference	Reference	Aβ − WMH+	0.021
			Aβ + WMH−	<0.001
			Aβ + WMH+	0.036
Aβ − WMH+	3.30	1.21–8.98	Aβ − WMH−	0.021
			Aβ + WMH−	0.358
			Aβ + WMH+	0.868
Aβ + WMH−	4.84	2.03–11.51	Aβ − WMH−	< 0.001
			Aβ − WMH+	0.358
			Aβ + WMH+	0.294
Aβ + WMH+	3.02	1.08–8.43	Aβ − WMH−	0.036
			Aβ − WMH+	0.868
			Aβ + WMH−	0.294

*A*β Amyloid-β, *WMH* White matter hyperintensities

Analyses are adjusted for demographics, study, and baseline diagnosis

### Post hoc analyses

Because both Aβ and WMH were associated with MTA, we tested the interaction between the two pathologies on MTA. General linear model analyses showed no interaction between Aβ and WMH on MTA, using dichotomous variables created with cutoff points (*p* = 0.770) or continuous variables (*p* = 0.631).

We repeated the main analyses after exclusion of subjects with CSF Aβ_1–42_ values 10% around the cutoff. The results remained similar after this exclusion. The results were also comparable after exclusion of the four subjects in the Aβ − WMH− group who progressed to FTD at follow-up, as well as when repeating the analyses using Aβ and tau for classification of AD profiles instead of only Aβ.

We conducted age sensitivity analyses in which we age-matched the Aβ/WMH groups by selecting only individuals between 64 and 79 years of age. Most results were similar to the original results. Results that were different are shown in Additional file 2. Associations that showed a similar direction but no longer reached significance because of a reduction in sample size (*p* values between 0.05 and 0.09) were considered unchanged.

Observed power calculations were done for the main analyses. For the comparisons of neurodegenerative markers (Table 2), the observed power ranged from 0.71 to 0.99. For the comparisons of cognitive performance and decline (Table 3), the observed baseline power ranged from 0.37 to 0.67, and for the slopes it ranged from 0.34 to 0.61. Regarding the comparisons in progression to dementia (Table 4), the observed power was 0.66.

## Discussion

We investigated the relation of Aβ and CVD pathology with markers of neurodegeneration and cognitive decline. We found that the neurodegeneration markers t-tau in CSF and MTA on MRI scans were associated with both Aβ and CVD, as well as that there was an additive association of the two pathologies on MTA. Decline of global cognition scores during follow-up was seen in both Aβ and CVD, but there was no additive or synergistic effect.

### Medial temporal lobe atrophy

The association between AD pathology and MTA has been well characterized in the literature. The first neuropathological changes underlying AD are thought to occur in the medial temporal lobe [37]. The relationship between MTA and CVD, however, is still somewhat controversial. Some studies have found that CVD was associated with MTA [12, 15, 38], whereas others did not find this relationship [7, 13]. Our results support an association between CVD and MTA, although we measured only one aspect of CVD (i.e., WMH). Interestingly, we found that MTA was most severe in the group with mixed Aβ/WMH pathology compared with the groups with a single form of pathology. In post hoc analyses, we found no interaction between Aβ and WMH on MTA, and therefore we conclude that WMH and Aβ are independent determinants of MTA severity and that when both are present, their effects are additive.

### Tau

Increased CSF t-tau was associated with both WMH and Aβ pathology, which is consistent with previous studies where t-tau was considered a measure of neuronal damage [14, 39]. Our finding that t-tau levels were also elevated in patients with WMH and no Aβ pathology contradicts a previous study in which researchers concluded that elevated t-tau levels in patients with vascular damage could be the result of coexisting Aβ pathology [40]. That elevated t-tau levels in the groups with one or both pathologies were found only in subjects with MCI is in line with previous work that strongly related tau to cognitive dysfunction [41]. p-tau was significantly increased only in the Aβ + WMH− group and only slightly increased in the Aβ + WMH+ group, supporting the hypothesis that this could be a specific biomarker for AD [39].

### Amyloid-β

We found similar levels of Aβ for the group with only amyloid pathology and the group with mixed Aβ/WMH pathology. This was in contrast to our expectations based on the literature, because we expected the group with mixed pathology to have a lower amyloid load (i.e., higher Aβ_1–42_ levels) [5, 6]. Possibly, the cognitive status of the investigated population (i.e., individuals with SCD or MCI vs. cognitively normal individuals) might play a role, in particular in combination with the method of measuring amyloid load (by CSF or amyloid positron emission tomography), because a study comparing these two methods showed that discordance was dependent on disease stage [42]. Further studies should be done to determine the associations of both factors with amyloid load in mixed AD/CVD patients. The suggestion of expanding the recently proposed “A/T/N” classification system with a vascular component would be valuable in addressing these and other research questions [43].

### Cognitive performance and decline

The decrease in performance in global cognition over time was similar for the three groups with pathology, indicating that WMH and Aβ pathology are drivers of cognitive decline. This is in line with findings derived from a previous study of cognitively normal individuals in which investigators also found that both pathologies contribute to cognitive decline [9]. However, in contrast to this previous study, we did not find any differences in cognitive trajectories between individuals with only Aβ pathology and those with mixed Aβ/WMH pathology. This may relate to the fact that we included subjects with SCD and MCI instead of cognitively normal subjects or to the type of cognitive measures used. Also, risk of progression to dementia during follow-up did not differ between WMH and Aβ, and having both pathologies simultaneously did not increase the risk any further.

### Strengths and limitations

This study has several limitations. First, using WMH as a marker of CVD can be seen as a limitation because we did not take other forms of CVD such as lacunar infarcts or cortical microbleeds into account. Although using only WMH reflects a method of defining vascular damage frequently used in clinical practice [44], this makes our findings less generalizable to CVD in general. Also, WMH are heterogeneous in their etiology and pathophysiology, and the underlying mechanisms causing WMH are not yet completely understood [45]. However, in an aging population such as we used in the present study, WMH are mostly considered a consequence of cerebral vascular damage [46, 47]. Second, our follow-up length ranged from 1 to 4 years, which might have been too short to detect differences in cognitive decline in nondemented individuals. Third, our sample was derived from different studies, which might have led to variability in the data, despite adjustment for study in all of the analyses. However, our multistudy design makes our findings more generalizable to other memory clinic settings. Fourth, a methodological consideration of this study was that our results were based on both subjects with SCD and subjects with MCI. Although we did examine the influence of the baseline diagnosis in all analyses and when needed adjusted for this and reported the differences, the smaller sample sizes when analyzing per diagnosis could have influenced the results. The smaller sample sizes in general could reflect a lack of statistical power, and therefore our results should be interpreted with caution and validated in future studies. Although observed power calculations should be interpreted with caution [48], we recommend that researchers in future studies make group sizes more balanced and include a larger number of complete follow-up visits, in particular for outcome measures with smaller effect sizes (e.g., executive functioning measures). The major strengths of this study were the longitudinal setup, the reflection of clinical practice, and the availability of different neurodegeneration markers to provide novel insights into the role of neurodegeneration in relation to AD and CVD.

## Conclusions

The findings of the present study may have implications for the diagnosis and prognosis of memory clinic patients but also for future scientific research. For clinicians, it is important to realize that MTA on MRI and elevated t-tau values in CSF may reflect underlying AD as well as CVD pathology, and that the effects of Aβ and WMH on MTA could be additive. On the basis of data derived from the present study, we conclude that the short-term cognitive prognosis of patients with SCD or patients with MCI with mixed amyloid/WMH pathology may be similar to that of patients with solely Aβ or WMH pathology. Future research with longer follow-up and a larger sample size is needed to confirm these findings and determine whether this is also the case when focusing on long-term prognosis.

## Additional files

Additional file 1: Scan parameters and MRI protocols used at each center. (DOCX 87 kb)

Additional file 2: Additional results in age-matched groups. Results that deviate in age sensitivity analyses from original findings. (DOCX 56 kb)

Additional file 3: Approval committee of each center. The ethics committee in each center that approved the data acquisition. (DOCX 84 kb)

## Abbreviations

AD: Alzheimer’s disease
APOE: Apolipoprotein E
ARMWS: Age-related white matter changes scale
Aβ: Amyloid-β
BBACL: BioBank Alzheimer Center Limburg
CSF: Cerebrospinal fluid
CVD: Cerebrovascular disease
DESCRIPA: Development of screening guidelines and criteria for predementia Alzheimer’s disease
FTD: Frontotemporal dementia
MCI: Mild cognitive impairment
MMSE: Mini Mental State Examination
MRI: Magnetic resonance imaging
MTA: Medial temporal lobe atrophy
p-tau: Phosphorylated tau
RAVLT: Rey Auditory Verbal Learning Test
SCD: Subjective cognitive decline
TMT-B: Trail Making Test part B
t-tau: Total tau
WMH: White matter hyperintensities
