# Correction to: Cerebrovascular and amyloid pathology in predementia stages: the relationship with neurodegeneration and cognitive decline

**DOI:** 10.1186/s13195-018-0391-x

**Published:** 2018-06-20

**Authors:** Isabelle Bos, Frans R. Verhey, Inez H. G. B. Ramakers, Heidi I. L. Jacobs, Hilkka Soininen, Yvonne Freund-Levi, Harald Hampel, Magda Tsolaki, Åsa K. Wallin, Mark A. van Buchem, Ania Oleksik, Marcel M. Verbeek, Marcel Olde Rikkert, Wiesje M. van der Flier, Philip Scheltens, Pauline Aalten, Pieter Jelle Visser, Stephanie J. B. Vos

**Affiliations:** 1 Department of Psychiatry and Neuropsychology, School of Mental Health and Neuroscience, Alzheimer Center Limburg, Maastricht University, Maastricht, The Netherlands; 2 Institute of Clinical Medicine, Neurology, University of Eastern Finland, Kuopio, Finland; 3 Neurocenter and Department of Neurology, Kuopio University Hospital, Kuopio, Finland; 4 Department of Neurobiology, Caring Sciences and Society (NVS), Karolinska University Hospital Huddinge, Stockholm, Sweden; 5 AXA Research Fund and UPMC Chair Sorbonne Universités, Université Pierre et Marie Curie (UPMC), Paris, France; 6 Institut du cerveau et de la moelle (ICM), Hôpital Pitié-Salpêtrière, Paris, France; 7 Memory and Dementia Center, 3rd Department of Neurology, Aristotle University of Thessaloniki, G Papanicolau” General Hospital, Thessaloniki, Greece; 8 Department of Clinical Sciences Malmö, Clinical Memory Research Unit, Lund University, Lund, Sweden; 9 Department of Radiology, Leiden University Medical Center, Leiden, The Netherlands; 10 Department of Gerontology and Geriatrics, Leiden University Medical Center, Leiden, The Netherlands; 11 Departments of Neurology and Laboratory Medicine, Donders Institute for Brain, Cognition and Behaviour, Radboud Alzheimer Center, Radboud University Medical Center, Nijmegen, The Netherlands; 12 Radboudumc Alzheimer Centre, Department of Geriatric Medicine, Radboud University Medical Center, Nijmegen, The Netherlands; 13 Department of Neurology, Alzheimer Centre, Neuroscience Campus Amsterdam, VU University Medical Center, Amsterdam, Netherlands

## Correction

Upon publication of this article [1], it was noticed that there were some inconsistencies in Tables 1, 2 and 3. Some of the superscript letters were incorrectly assigned. Please see below the correct tables:

**Table 1:** Comparisons of baseline and follow-up characteristics by Aβ and WMH status

	Aβ- WMH-	Aβ- WMH+	Aβ + WMH-	Aβ + WMH+
	*n* = 140	*n* = 39	*n* = 63	*n* = 29
Baseline characteristics				
Age	61.7 (8.3)^B,C,D^	71.3 (7.7)^A,C^	66.7 (7.8)^A,B,D^	74.1 (5.0)^A,C^
Female, n	94 (67)^C^	23 (59)	32 (51)^A^	16 (55)
Education in years	10.9 (3.1)	11.9 (3.3)	11.1 (3.1)	10.3 (2.9)
Hypertension, n^*^	43 (34)	9 (25)	15 (25)	9 (32)
Obesity, n^*^	15 (14)	3 (11)	4 (8)	4 (21)
Diabetes, n^*^	16 (21)	3 (15)	3 (7)	5 (28)
APOE-ε4 carrier, n^*^	33 (51)^B^	5 (24)^A,C,D^	29 (62)^B^	10 (56)^B^
Diagnosis MCI, n	70 (50)^D^	21 (54)^D^	40 (64)	22 (76)^A,B^
amnestic MCI (% within MCI group)	40 (57)	15 (71)	27 (68)	17 (77)
non-amnestic MCI (% within MCI group)	30 (43)	6 (29)	13 (33)	5 (23)
CSF Aβ 1–42, pg/ml	973.6 (312.0)^C,D^	885.0 (242.0)^C,D^	404.3 (102.6)^A,B^	419.3 (97.2)^A,B^
White matter hyperintensities^†^	0.7 (0.5)^B,D^	2.3 (0.4)^A,C^	0.8 (0.4)^B,D^	2.4 (0.5)^A,C^
Follow-up characteristics				
Follow-up time	2.1 (1.5)	2.2 (1.3)	2.1 (1.2)	2.4 (1.2)
Time to progression to dementia	1.3 (0.5)^B^	2.0 (0.7)^A^	1.7 (0.7)	2.1 (1.2)
Progression to dementia, n	8 (6)^B,C,D^	9 (23)^A^	18 (29)^A^	11 (38)^A^
- AD-type dementia, n	2 (1)^B,C,D^	7 (18)^A^	18 (29)^A^	10 (35)^A^
- Vascular dementia, n	0 (0)	2 (5)	0 (0)	1 (3)
- Frontotemporal dementia, n	4 (3)	0 (0)	0 (0)	0 (0)
- Lewy Body dementia, n	1 (1)	0 (0)	0 (0)	0 (0)
- Dementia with unknown etiology, n	1 (1)	0 (0)	0 (0)	0 (0)

Results are mean (SD) for continuous variables or frequency (%). Hypertension, obesity, diabetes and APOE ε4 genotype were only available in a subgroup of the sample

Abbreviations: *Aβ* amyloid-beta, *AD* Alzheimer’s disease, *APOE* Apolipoprotein E, *MCI* mild cognitive impairment

^†^WMH measured by the Fazekas scale, range 0–3

^A^*p* < 0.05 compared to Aβ- WMH-

^B^*p* < 0.05 compared to Aβ- WMH+

^C^*p* < 0.05 compared to Aβ + WMH-

^D^*p* < 0.05 compared to Aβ + WMH+

**Table 2:** Values of neurodegenerative markers by Aβ/WMH groups

	Aβ- WMH-	Aβ- WMH+	Aβ + WMH-	Aβ + WMH+
Neurodegeneration markers	n = 140	n = 39	n = 63	n = 29
MTA score	1.2 (1.2)^B,C,D^	2.6 (1.6)^A,D^	2.1 (1.6)^A,D^	3.4 (1.8)^A,B,C^
MTA abnormal, n	62 (45)^B,C,D^	32 (82)^A^	41 (67)^A,D^	26 (93)^A,C^
P-tau, pg/ml	54.5 (27.7)^C^	63.2 (29.3)	77.0 (56.3)^A^	65.2 (38.2)
P-tau abnormal, n	53 (38)^C^	22 (58)	45 (71)^A^	15 (52)
T-tau, pg/ml	314.7 (202.0)^B,C,D^	438.4 (248.0)^A^	499.3 (413.8)^A^	426.2 (275.2)^A^
T-tau abnormal, n	36 (26)^B,C,D^	20 (53)^A^	36 (57)^A^	14 (48)^A^

Results are mean (SD) and number (%). All analyses were adjusted for study, baseline diagnosis and demographics

*Abbreviations*: *Aβ* amyloid-beta, *MTA* medial temporal lobe atrophy, *P-tau* phosphorylated tau, *T-tau* Total tau, *WMH* white matter hyperintensities

^A^*p* < 0.05 compared to Aβ- WMH-

^B^*p* < 0.05 compared to Aβ- WMH+.

^C^p < 0.05 compared to Aβ + WMH-.

^D^p < 0.05 compared to Aβ + WMH+.

**Table 3:** Cognitive performance and decline by Aβ/WMH groups

		Aβ- WMH-	Aβ- WMH+	Aβ + WMH-	Aβ + WMH+
MMSE^*^	n	140	39	62	27
	Baseline	27.79 (27.39, 28.19)	27.52 (26.83, 28.21)	27.20 (26.62, 27.78)	27.40 (26.54, 28.25)
	Slope	−0.01 (− 0.15, 0.12)	− 0.29 (− 0.55, − 0.02)	−0.22 (− 0.44, − 0.01)	− 0.31 (− 0.62, 0.00)
Memory delayed recall z-score	n	133	37	58	27
	Baseline	−0.48 (− 0.72, − 0.24)^B,C,D^	−1.04 (− 1.48, − 0.61)^A^	−1.04 (− 1.41, − 0.68)^A^	−1.33 (− 1.86, − 0.80)^A^
	Slope	0.05 (− 0.03, 0.13)	0.02 (− 0.12, 0.17)	0.02 (− 0.11, 0.14)	−0.07 (− 0.24, 0.09)
Executive functioning z-score	n	130	37	60	24
	Baseline	−0.48 (− 0.76, − 0.21)	−0.41 (− 0.92, 0.09)	−0.78 (− 1.18, − 0.37)	−1.12 (− 1.73, − 0.50)
	Slope	0.06 (− 0.02, 0.13)	−0.00 (− 0.15, 0.15)	−0.03 (− 0.16, 0.10)	−0.04 (− 0.23, 0.15)

Results are mean (95% confidence interval). Bold slope estimates = *p* < 0.05. All analyses were adjusted for study. The analyses on MMSE scores were also corrected for demographics and baseline diagnosis

*Abbreviations*: *Aβ* amyloid-beta, *MMSE* mini mental state examination, *WMH* white matter Hyperintensities

^A^p < 0.05 compared to Aβ- WMH-

^B^p < 0.05 compared to Aβ- WMH+.

^C^p < 0.05 compared to Aβ + WMH-.

^D^p < 0.05 compared to Aβ + WMH+.
