# Supplementary material for: Cerebrovascular and amyloid pathology in predementia stages: the relationship with neurodegeneration and cognitive decline
Source: Alzheimers Res Ther. 2017 Dec 29;9:101. doi: 10.1186/s13195-017-0328-9 (PMC5747152; doi:10.1186/s13195-017-0328-9)
Supplement: Supplementary file 1 — Scan parameters and MRI protocols used at each center. (DOCX 87 kb) [file 13195_2017_328_MOESM1_ESM.docx]

**Scan parameters and MRI protocols used at each centre.**

**DESCRIPA**

CENTER HUDDINGE - STOCKHOLM

Siemens Avanto 1.5 T, 21 slices, FOV 220 mm, FOV phase 87.1, distance factor 30, phase R>L, slice thickness 5.0 mm, TE: 96 ms, TR: 4000 ms, flip angle 150°, number of averages 1.

Siemens Symphony 1.5 T, 21 slices, FOV 220 mm, FOV phase 75.0, distance factor 30, phase R>L, slice thickness 5.0 mm, TE: 99 ms, TR: 4100 ms, flip angle 150°, number of averages 2.

CENTER KUOPIO

Siemens Vision 1.5 T, T1 3D-scan, MPRAGE OBL; COR>TRA, FOV 250, mat 256x256, 128 slices, TR 9.7ms, TE 4 ms, Slice th 2.0mm, no slice gap, flip angle 12°

CENTER MALMO

Siemens Sonata 1.5 T, MPRAGE + lmpr-cor, 144 slices, FOV 250 mm, phase R>L, TR 1970, TE 3.93, distance factor 50, slice thickness 1.5 mm, flip angle 15°.

CENTER MUNICH

Siemens Magnetom Vision; 1.5 T; MPRAGE; Slice thickness 1.05 mm, TR 11.4, TE 4.4, TI 300; FOV 256*256; Flip angle 8°; number of averages 1.

CENTER THESSALONIKI

Siemens Expert Plus unit 1.0 T, 3D-MPR: 15 (TR) ,7 TE ,8 FLIP ANG., 250 (Slabth), 1,49Ef thick, 168 Partitions, 250 FOV,256x192 Matrix, 1 Aquis. ,ACQ TIME 10,21min

CENTER MAASTRICHT

Philip NT, 1.5 T Gyroscan: T1-weighted images obtained in the coronal plane using a 3D-gradient fast field echo (FFE) sequence. TR = 35 ms, TE = 7 ms, FA= 35, FOV= 240 mm, slice thickness = 1.5 mm, matrix size = 256x256, voxelsize = 0.94mm x 0.94mm x 1.5 mm.

CENTER MANNHEIM

Siemens Medical solution Magnetom, Vision plus 1.5 Tesla: T1 MPR 30, TR=11.4 ms, TE=4.4 ms, flipangle=15, FoV=256mm, format 8/8, slices=162, no gap.

CENTER VUMC

Siemens Magnetom Impact Expert 1.0 T, 3D scan, 168 slices, FOV 250 mm, matrix 256 × 256; slice thickness 1.5 mm, TE: 7 ms, TR: 15 ms, TI 300 ms, ﬂip angle 15°.

**LeARN**

CENTER LEIDEN

Philips Achieva, 3.0 T, 3D scan, 180 slices; matrix: 288 x 288; voxel size: 0.7778 x 0.7778 x 1.00; TE: 4.6 ms; TR: 9.8 ms; flip angle: 8°.

CENTER NIJMEGEN

Siemens TrioTim 3.0 T, 3D scan; 192 slices; matrix: 256 x 256; voxel size: 1.00 x 1.00 x 1.00; TE: 4.7 ms; TR: 2300 ms; TI: 1100 ms; flip angle: 90°.

CENTER VUMC

GE Signa HDxt 3.0 T; 3D-scan; 176 slices; matrix: 256 x 256; voxel size: 0.94 x 0.94 x 1.00; TE: 3.0 ms; TR: 7.8 ms; TI: 450 ms; flip angle: 90°

CENTER MAASTRICHT + additional subjects from BBACL

Philips Achieva 3.0 T; 3D scan; 180 slices; 240 x 240; voxel size: 1.00 x 1.00 x 1.00; TE: 3.7 ms; TR: 8.2 ms; flip angle: 8°.
