# Supplementary material for: Cerebrovascular and amyloid pathology in predementia stages: the relationship with neurodegeneration and cognitive decline
Source: Alzheimers Res Ther. 2017 Dec 29;9:101. doi: 10.1186/s13195-017-0328-9 (PMC5747152; doi:10.1186/s13195-017-0328-9)
Supplement: Supplementary file 2 — Additional results in age-matched groups. Results that deviate in age sensitivity analyses from original findings. (DOCX 56 kb) [file 13195_2017_328_MOESM2_ESM.docx]

|  | | | | | |
| --- | --- | --- | --- | --- | --- |
|  | | **Aβ- WMH-** | **Aβ- WMH+** | **Aβ+ WMH-** | **Aβ+ WMH+** |
|  | | n=52 | n=26 | n=43 | n=22 |
| Age | | 70.3 (4.3) | 70.4 (4.8) | 70.5 (3.8) | 72.8 (3.9) |
| **Neurodegeneration markers** | | |  |  |  |
| MTA score | 1.6 (1.4)^B,D^ | | 2.6 (1.5)^A^ | 2.2 (1.7) | 2.9 (1.6)^A^ |
| MTA abnormal, n | 31 (62%)^D^ | | 21 (81%) | 22 (67%)^D^ | 19 (91%)^A,C^ |
| **Cognition** |  | |  |  |  |
| MMSE – Slope | -0.10 (-0.36, 0.16)^C^ | | -0.35 (-0.73, 0.03) | **-0.65 (-1.03, -0.26)**^A^ | **-0.45 (-0.87, -0.03)** |
| Memory - Slope | 0.09 (-0.02, 0.20)^D^ | | 0.06 (-0.10, 0.23) | -0.11 (-0.29, 0.06) | -0.12 (-0.29, 0.06)^A^ |
| Results are mean (SD or 95% CI) or frequency (%). Abbreviations: Aβ = amyloid-beta, MMSE= Mini Mental State Examination, WMH= White Matter Hyperintensities. Bold slope estimates = p<0.05. ^A^ p<0.05 compared to Aβ- WMH-, ^B^ p<0.05 compared to Aβ- WMH+, ^C^ p<0.05 compared to Aβ+ WMH-, ^D^ p<0.05 compared to Aβ+ WMH+. All analyses were adjusted for study. The analyses on MMSE scores were also corrected for demographics and baseline diagnosis. | | | | | |

**Additional results in age-matched groups**
