# Supplementary material for: Cerebrovascular and amyloid pathology in predementia stages: the relationship with neurodegeneration and cognitive decline
Source: Alzheimers Res Ther. 2017 Dec 29;9:101. doi: 10.1186/s13195-017-0328-9 (PMC5747152; doi:10.1186/s13195-017-0328-9)
Supplement: Supplementary file 3 — Approval committee of each center. The ethics committee in each center that approved the data acquisition. (DOCX 84 kb) [file 13195_2017_328_MOESM3_ESM.docx]

| **Center** | **Country** | **Approval Committee** |
| --- | --- | --- |
| *BBACL* |  |  |
| Maastricht University Medical Center, Maastricht | The Netherlands | Medical ethical committee Maastricht University Medical Center |
| *DESCRIPA* |  |  |
| Karolinska University Hospital, Huddinge | Sweden | Regional Ethical Review Board Stockholm |
| Kuopio University Hospital, Kuopio | Finland | Research Ethics Committee of the Northern Savo Hospital District |
| Maastricht University Medical Center, Maastricht | The Netherlands | Medical ethical committee Maastricht University Medical Center |
| Lund University, Malmö | Sweden | Ethics Committee, Lund University Hospital |
| Aristotle University, Thessaloniki | Greece | Aristotle University of Thessaloniki Medical School Ethics Committee |
| Ludwig-Maximilian University, Munich | Germany | Ethical Committee Maximilian University Munich |
| VU Medical Center, Amsterdam | The Netherlands | Medical ethics committee VU Medical Center |
| *LeARN* |  |  |
| Leiden University Medical Center, Leiden | The Netherlands | Medical ethical committee Leiden University Medical Center |
| Maastricht University Medical Center, Maastricht | The Netherlands | Medical ethical committee Maastricht University Medical Center |
| VU University Medical Center, Amsterdam | The Netherlands | Medical ethical committee VU Medical Center |
| Radboud University Nijmegen Medical Centre, Nijmegen | The Netherlands | Committee on Research Involving Human Subjects region Arnhem-Nijmegen |
|  |  |  |

**Approval committee in each centre**
